# Assessment of Hyperactive Reflexes in Patients with Spinal Cord Injury

**DOI:** 10.1155/2015/149875

**Published:** 2015-01-15

**Authors:** Dali Xu, Xin Guo, Chung-Yong Yang, Li-Qun Zhang

**Affiliations:** ^1^Rehabilitation Institute of Chicago, 345 E. Superior Street, Room 1406, Chicago, IL 60611, USA; ^2^Department of Automation, Hebei University of Technology, Tianjin 300401, China; ^3^Departments of Biomedical Engineering, Northwestern University, Chicago, IL 60611, USA; ^4^Departments of Orthopaedic Surgery, Northwestern University, Chicago, IL 60611, USA; ^5^Departments of Physical Medicine and Rehabilitation, Northwestern University, Chicago, IL 60611, USA; ^6^Department of Orthopaedic Surgery, Northshore University HealthSystem, Evanston, IL 60201, USA

## Abstract

Hyperactive reflexes are commonly observed in patients with spinal cord injury (SCI) but there is a lack of convenient and quantitative characterizations. Patellar tendon reflexes were examined in nine SCI patients and ten healthy control subjects by tapping the tendon using a hand-held instrumented hammer at various knee flexion angles, and the tapping force, quadriceps EMG, and knee extension torque were measured to characterize patellar tendon reflexes quantitatively in terms of the tendon reflex gain (*G*_tr_), contraction rate (*R*_*c*_), and reflex loop time delay (*t*_*d*_). It was found that there are significant increases in *G*_tr_ and *R*_*c*_ and decrease in *t*_*d*_ in patients with spinal cord injury as compared to the controls (*P* < 0.05). This study presented a convenient and quantitative method to evaluate reflex excitability and muscle contraction dynamics. With proper simplifications, it can potentially be used for quantitative diagnosis and outcome evaluations of hyperreflexia in clinical settings.

## 1. Introduction

Brain lesions or impaired spinal cord induces an interruption of corticospinal and other descending pathways, which influence the function of the reflex arc, disrupt the remaining functional use of muscles, and impede motion. Uncontrollable sensory-motor hyperexcitability from the stretch of the impaired limb, so called spastic hypertonia [1, 2], may be accompanied by structural changes of muscle fibers and connective tissue. It may also result in a reduction in joint range of motion and lead to clinical contracture [3]. Many therapeutic paradigms, such as antispastic medication, physical modalities, botulinum toxin injection, and intrathecal baclofen pumps, were developed and applied for the purpose of reducing spasticity and improving function [4–10]. Despite intensive research, the mechanisms of causing those abnormal phenomena, such as hyperexcitability and contractures, in neurological disorders including SCI are not well characterized [11].

Spastic muscle hypertonus is considered attributable to increased stretch reflex activity though passive mechanical properties may also play a role [12]. The increased resistance to passive movement in a spastic limb can be due to nonreflex changes like increased muscle stiffness with reduced joint range of motion as well as reflexive changes like hyperactive reflexes [12–14]. Reflex and nonreflex-mediated contributions to the increased resistance need to be separated in order to evaluate and understand the mechanisms underlying spasticity. Furthermore, it is not clear whether the hyperactive reflexes associated with spasticity are due to an increase in reflex gain or a decrease in reflex threshold [15–18]. To assess severity of the spastic state and characterize its properties following SCI, it is important to evaluate hyperactive reflexes quantitatively, including reflex gain, contraction rate, and threshold in SCI survivors. Deep tendon reflex scale is commonly used to characterize hyperactive reflexes and modified Ashworth scale is used to characterize spasticity in clinical practice. These manual evaluations are convenient but lack accurate measurements for clinical diagnosis and treatment outcome evaluations [19]. Tendon tapping with a custom hammer, which had a load cell mounted at its head to measure tapping force, was used to evaluate hyperactive reflex changes more quantitatively in multiple sclerosis and stroke [18, 20].

The purpose of the study was to quantitatively characterize the hyperactive tendon reflexes associated with spastic hypertonia in SCI. Patellar tendon tapping force was taken as the system input and reflex-mediated muscle EMG and joint torque as the system outputs. The system impulse response was obtained through system identification and characterized by the tendon reflex gain, contraction rate, reflex threshold in tapping force, and reflex loop delay. The experiment was conducted under isometric condition so that nonreflex contributions were largely eliminated. The hypothesis was that hyperactive reflexes in SCI were associated with significantly increased reflex gain, contraction rate, and decreased reflex threshold and reflex loop delay.

## 2. Method

### 2.1. Subject Selection

Nine patients with spinal cord injury (Table 1) (age: 32.8 ± 6.7 years, height: 171.3 ± 4.5 cm, weight: 76.6 ± 15.8 kg, all males) and ten healthy subjects with no prior history of neurological disorders (age: 38.1 ± 5.9 years, height: 175.6 ± 6.6 cm, weight: 66.7 ± 7.3 kg, 8 males and 2 females) participated in this study. Each SCI survivor was examined at the beginning of the experiment using the clinical tendon reflex scale ranging from 0 to 4 with 0 for no response, 1 for low average, 2 for average normal, 3 for brisk more than average, and 4 for hyperactive and association with clonus. All subjects gave informed consent before participating in the study, which was approved by the Institutional Review Board at Northwestern University.

### 2.2. Experimental Procedures

**Figure 1:**
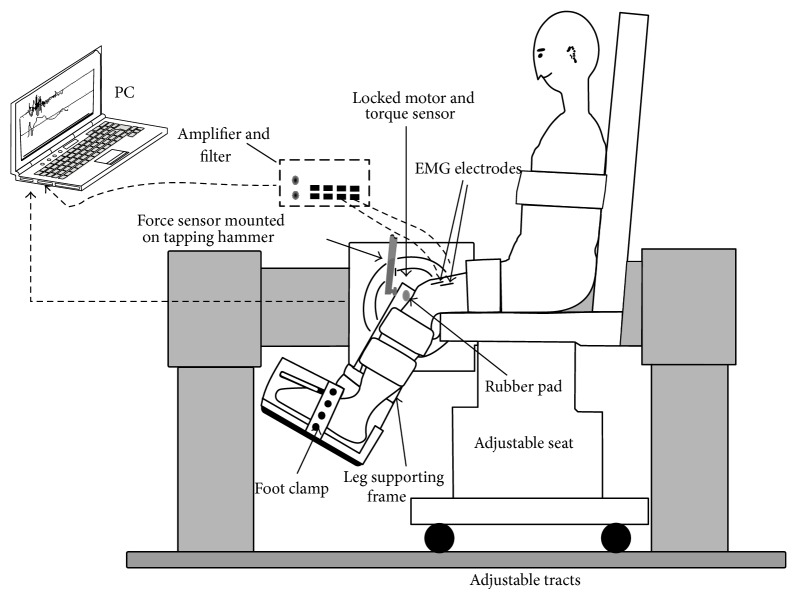
Experimental setup for the patellar tendon tapping. The motor was mounted on the supporting frame with the motor shaft aligned with the knee flexion axis. A 6-axis force/torque sensor was mounted between the motor shaft and the aluminum beam to measure reflex knee extension torque. The cast was fixed to the aluminum beam through the coupling. The motor was fixed at a selected joint flexion angle to eliminate knee motion. The patellar tendon was tapped using an instrumented tendon hammer with a semiconductor load cell mounted at tip of the hammer to measure tapping force.

The subject sat upright with the thigh and trunk strapped to a custom-designed seat (Figure 1). The ankle was mounted onto one end of an aluminum beam, and the other end of the beam was mounted onto a motor shaft through a torque sensor that measured the reflex torque response. The motor was locked at the selected knee flexion angle during the experiment, restricting the knee at an isometric condition.

Using a traditional tendon reflex mallet, the most sensitive spot on quadriceps tendon with the strongest reflex response was located. A hemisphere self-adhesive rubber pad with a diameter of 10 mm was pressed onto the quadriceps tendon at the most sensitive spot. An instrumented tendon hammer with a force sensor mounted at its head was used to tap the rubber pad. The flat impact surface of the instrumented tendon hammer hits the dome-shaped rubber pad, which made the tapping force transmission onto the tendon more accurate and consistent, reducing variations of the tendon reflexes [10, 18].

During the experiment, the subject was seated comfortably and was asked to fully relax and not to react and anticipate the taps. If the subject felt inclined to move or change the posture, we would wait until he or she settled down again. At the beginning, the tapping force was adjusted so that a quadriceps muscle contraction was evoked by visual inspection. The quadriceps tendon was then tapped at approximately that level about seven times during a trial, with a random interval averaging about 2.5 seconds. Three trial sequences were collected. The tendon tapping force, rectus femoris, vastus lateralis and vastus medialis EMG signals, and knee joint extension torque were sampled by a computer at 500 Hz after low-pass filtering (8th-order Butterworth filter at 230 Hz cutoff).

### 2.3. Data Processing

The sampled tendon tapping force, quadriceps EMG, and knee extension torque signals were low-pass filtered and edited interactively with a cutoff frequency of 150 Hz. Knee extension torque and rectus femoris EMG signals were inspected to see whether there was any voluntary contraction. If so, the relevant taps were excluded. The stretch reflex loop delay (*t*_*d*_) was determined from the onset of the tapping force to the onset of the reflex-mediated torque. Multiple taps were used to derive a more reliable estimate. EMG and torque signals were then segmented into multiple taps, aligned by the tapping force peak moment. Each data segment was about 670 msec long, started from 70 msec before the tapping force peak, and ended 600 msec after the peak.

### 2.4. System Impulse Response

Since the reflex-mediated torque is induced by the tendon tapping force and varied with the tapping force, it is appropriate to treat them as output and input of the tendon reflex system, respectively [10, 21]. The system impulse response was used to characterize the reflex-mediated torque as the output of a system excited by the tendon tapping force. The impulse response (the input-output relationship) was identified from the experimental data as follows. Since the tapping force was rather brief, it could be approximated as a pulse. Therefore, the impulse response was approximated as the reflex-mediated torque response scaled by the area of the corresponding tapping force pulse [18].

### 2.5. Parameters Characterizing Tendon Reflex Dynamics

Several physiologically meaningful parameters were used to characterize the impulse response of the tendon reflex system [18]. The first was the tendon reflex gain (*G*_tr⁡_). Within a certain range, the reflex-mediated torque varied with the tendon tapping force, and a stronger tapping force elicited a stronger reflex-mediated torque. In system analysis, *G*_tr⁡_ was the gain measure of the tendon reflex system, from the tendon tapping force as the system input (in unit of N) to the joint torque as the system output (in unit of Nm). The ratio of system output over system input is Nm/N = m. The unit here for *G*_tr⁡_ is therefore meter (m) or centimeter (cm). We can consider it as a lever arm, for a given amount of tapping force, how much torque can be generated at the joint.

The contraction rate (*R*_*c*_) characterized the slope of the ascending segment of the impulse response (calculated over the period from the onset to the peak instant of the impulse response). Contraction rate characterized the muscle contraction dynamics with a unit of m/sec. Reflex responses may be evoked by a weaker stimulus in a spastic limb than in a normal limb. Therefore, a useful measure of hyperactive reflexes was the threshold in tendon tapping force (*f*_th_) for evoking reflex responses. Since the tendon was tapped repeatedly in the experiment just above the threshold, the averaged peak tapping force was used as the threshold in tapping force. Finally, the reflex loop delay (*t*_*d*_) was characterized quantitatively as the delay from the start of the tapping force to the onset of the reflex-mediated torque response, which was also shown in the impulse response.

### 2.6. Statistical Analysis

A repeated measures design examined tendon tapping and reflex responses across different knee joint positions. Comparisons of the *G*_tr⁡_, *R*_*c*_, *f*_th_, and *t*_*d*_ between the two participant groups across the four different knee joint angles were made using the two-way repeated measures analysis of variance (ANOVA). The significance level was chosen at *α* = 0.05.

## 3. Results

### 3.1. Typical Tendon Reflexes in SCI and Control Groups

**Figure 2:**
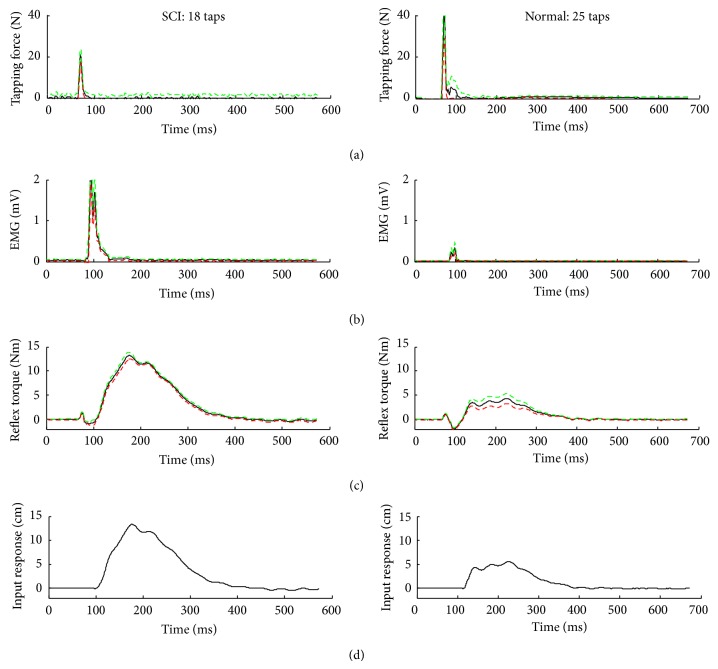
Representative tendon tapping results over multiple taps of the quadriceps tendon with the knee joint at 45° flexion. The black solid lines represent means for each variable. The red dashed lines and the green dashed lines represent plus and minus one standard deviation, respectively. The first row of the plots from both columns was tapping force, the second row of the plots was EMG, the third row of the plots was the reflex torque, and the last row of the plots was the impulse response.

Compared to the control group, SCI survivors showed different neuromuscular dynamics in tendon reflexes. As shown in the representative cases (Figure 2), the peak tapping force for the SCI survivor (22 ± 2 N, mean ± standard deviation) was lower than that (41 ± 4 N, mean ± standard deviation) in the control group without any neurological disorder. On the other hand, the reflex-mediated EMG response and knee extension torque in the SCI survivor were much higher and changed more quickly than their counterparts in the control without any neurological disorder.

### 3.2. Impulse Response of the Tendon Reflexes

The impulse responses characterized the dynamic relationship between the tapping force and reflex-mediated torque response in the point of view of system analysis. Compared to the control, the spastic leg showed a much stronger tendon reflex impulse response with much higher amplitude, more quickly increased amplitude, indicating stronger and quicker reflex responses associated with hyperactive reflexes.

**Figure 3:**
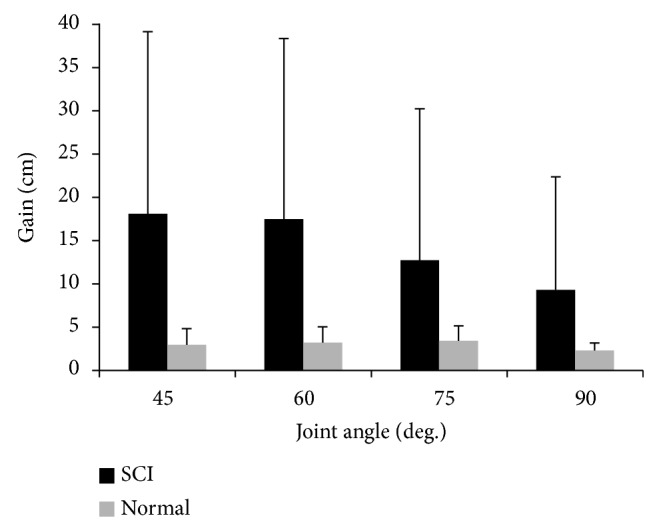
Comparison of reflex gain between the SCI group and the control group.

*Tendon Reflex Gain.* The tendon reflex gain *G*_tr⁡_ in SCI survivors was much higher than that in controls (Figure 3), and there was significant difference between the two groups (*F*_(1,8)_ = 5.777, *P* = 0.044). The mean and standard deviation of the tendon reflex gain of the SCI group versus the reflex gain of the control group were 18.09 (±21.1) cm versus 2.9 (±1.86) cm at 45° knee flexion, 17.47 (±20.89) cm versus 3.2 (±1.83) cm at 60° knee flexion, 12.72 (±17.51) cm versus 3.42 (±1.71) cm at 75° knee flexion, and 10.07 (±13.09) cm versus 2.29 (±0.88) cm at 90° knee flexion, respectively (Figure 3).

**Figure 4:**
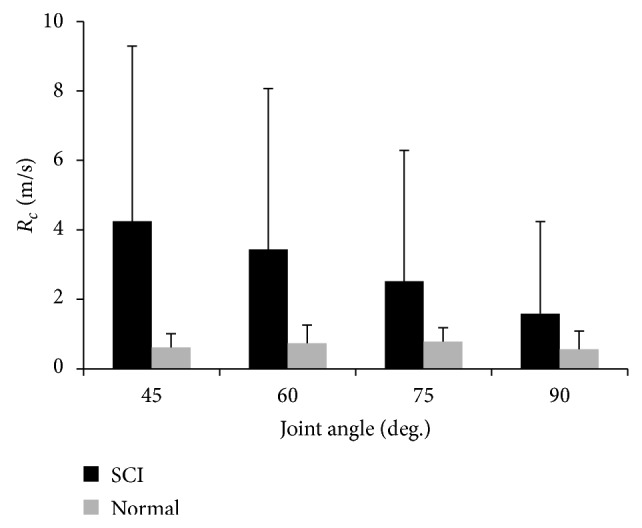
Comparison of contraction rate between the SCI group and the control group.

*Tendon Reflex Contraction Rate.* The ANOVA procedures with repeated measures showed that the Rc of the reflex from the SCI group was significantly higher than that from the control group (see Figure 4) across the different angles (*F*_(1,8)_ = 10.765, *P* = 0.011). The mean values for the contraction rate of the tendon reflex were 4.2 (±6.05) m/s versus 0.62 (±0.39) m/s at the 45° knee flexion, 3.44 (±5.6) m/s versus 0.74 (±0.52) m/s at 60° knee flexion, 2.5 (±3.8) m/s versus 0.78 (±0.41) m/s at 75° knee flexion, and 1.59 (±2.65) m/s versus 0.57 (±0.52) m/s at 90° knee flexion.

**Figure 5:**
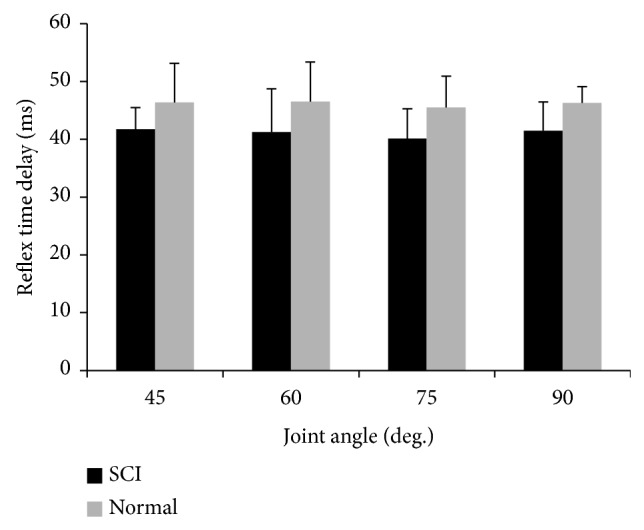
Comparison of reflex time delay between the SCI group and the control group.

*Reflex Time Delay.* There was a significant difference of the reflex loop delay between the SCI group and the control group (*F*_(1,8)_ = 12.742, *P* = 0.007). The mean values of the reflex loop delay for the SCI survivor versus control group at 45, 60, 75, and 90° knee flexion were 41.73 (±6.7) msec versus 46.37 (±3.7) msec, 41.23 (±6.87) msec versus 46.5 (±7.5) msec, 40.08 (±5.4) msec versus 45.49 (±5.2) msec, and 41.48 (±2.8) msec versus 46.26 (±4.97) msec, respectively (Figure 5). However, there were no significant differences in the *t*_*d*_ between different joint angles. This indicates that the reflex loop delay is not sensitive to changes in joint angles among the SCI and control groups.

*Tapping Force Threshold.* A tap on the most sensitive spot of the patellar tendon elicited a reflex contraction of the quadriceps, which then generated a knee extension torque. Compared with spastic limbs in SCI patients, there was no significant difference in the tapping force (*F*_(1,8)_ = 0.692, *P* = 0.43) needed to evoke reflex responses between the SCI group and the control group across the different joint angles.

## 4. Discussion

Spastic hypertonia is associated with uncontrollable sensory-motor hyperexcitability due to the lack of upper motor neuron control from the central nervous system. Reflex responses can be extremely variable, especially in patients with neurological disorders, especially for spasticity. It suggests that spasticity is a complex phenomenon involving reflex and nonreflex components, each of which needs to be quantified in relation to its corresponding clinical facet in order to explain and understand the multifaceted clinical features of spasticity [12, 22–24]. Focusing on the hyperactive reflexes, tendon reflexes were evaluated in this study under the isometric condition, which effectively minimized the mechanical contributions of joint stiffness, viscosity, and limb inertia. Therefore, the reflex contribution was manifested and readily separated from the intrinsic and passive contributions to joint torque. It was employed in relevant studies to make accurate measurements of both taps to the tendon and the reflex-mediated responses and to characterize their dynamic relationship in terms of tendon reflex gain, contraction rate, and tapping force threshold [12, 18, 25].

The tendon reflex gain was the system gain calculated through system identification, relating the input of tapping force to the output of the reflex-mediated torque response. The system parameters quantified the input and output simultaneously and gave more reliable measures than did the input or output parameters alone [18]. The results showed markedly increased system gain, contraction rate, and decreased reflex loop delay. The quadriceps tendon reflex was much more excitable in SCI survivors than that in controls, which was consistent with previous findings in the literature [10, 21, 26]. The increased tendon reflex gain and contraction rate could be due to a higher level of *α*-motoneuron activation, which evoked quicker and stronger muscle contraction. Possible mechanisms for the higher activation of *α*-motoneurons in SCI survivors include increased spindle afferent discharge rates from group Ia muscle spindle afferents, which might be related to tighter mechanical coupling transmitting stronger stretch from tendon tapping to the spindles, remaining fusimotor tone due to *γ*-motoneuron dysfunction, and increased excitatory presynaptic input so that excitatory postsynaptic potential and motoneuronal excitability increased. It was also possible that the inhibitory synaptic input might be reduced; namely, presynaptic inhibition initiated by descending fiber input was reduced due to lack of the influence of the corticospinal control.

There was a shorter latency of the reflex loop delay in patients with SCI in this study, which could be due to heightened state of spastic muscles with quicker development of muscle force and/or due to reduction in the presynaptic inhibition between Ia afferent and *α*-motoneurons under altered upper motor neuron control.

**Figure 6:**
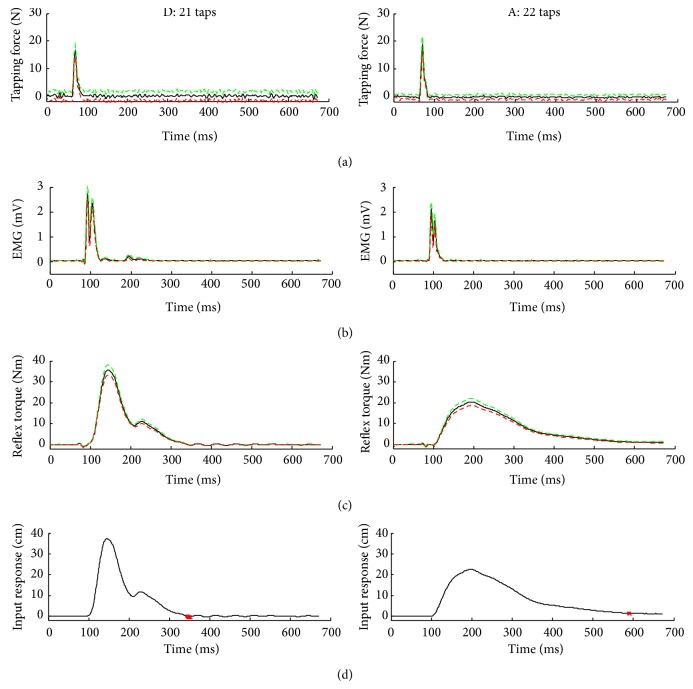
Comparison of tendon tapping results between two SCI individuals with spastic hypertonia. Subject D in the left olumn had an extremely strong reflex-mediated torque response but it damped out quickly, dropped to zero at 350 milliseconds (red dot). Subject A in right column, in contrast, had a less strong reflex-mediated torque response but it lasted much longer, dropped to zero at 600 milliseconds (red dot), indicating stronger tonic reflex response.

Interestingly, different patterns of hyperactive reflex dynamics were seen in patients with SCI. Examples of impulse responses from two different SCI patients are shown in Figure 6. For subject D, the reflex-mediated torque was extremely strong but was damped out quickly after about 350 milliseconds, while subject A had less strong but longer sustaining contraction than that of subject D, which was reduced to zero at about 600 milliseconds, indicating a stronger tonic reflex component (see the red dots in the last row of the two impulse responses in Figure 6). It is possible that there was not only monosynaptic but also oligosynaptic contributions to the reflex response [27]. It is also possible that both group Ia and group II afferents contributed to the reflex-mediated reflex response with the afferent potential from group II propagated slower than group Ia and thus the *α*-motoneurons had longer excitation. On the other hand, the reflex-mediated torque response from the control group was generally weaker, short in duration, and with more consistent pattern, as compared to patients with SCI. It suggests that because of the spinal cord and nerve roots being damaged in different locations and different levels, the phasic and tonic reflex components may be altered in different ways. The further studies are necessary in SCI survivors.

Of note is that excitability of stretch reflex may decrease when repetitive stimuli are used. Hultborn et al. [28] and Schindler-Ivens and Shields [29] reported that the soleus H-reflex amplitude was decreased with increasing stimulation frequency in patients and healthy participants. Grey et al. [30] found that H-reflexes were depressed by postactivation depression to a much greater extent than stretch reflexes in both healthy and spastic participants for short intervals of repetitive stimuli, especially in healthy subjects if the interval between stimuli was less than 10 seconds. In this tendon reflex study, repeated tendon tapping may similarly induce postactivation depression and it may affect the SCI and healthy populations differently. However, in this study, tendon tapping was done in trials of 20 sec long with about 6 to 7 taps per trial. There was a rest period between successive trials so that each trial started with fresh taps and only several brief taps were delivered within each trial, which helped reduce the effect of postactivation depression. Furthermore, there was no limb movement during tendon tapping in this study, which might also reduce postactivation depression. Still, postactivation depression might affect the results of this study. In various related studies, different intervals between taps have been used. Chandrasekhar et al. [31] used 10 seconds for a tapping interval in a tendon reflex study. Chardon et al. [32] used five brief taps at an interval of 2.5 seconds. The interval in this study was about 3 seconds, which is similar to previous studies [10, 18, 20, 33].

A limitation of the study was that the subject sample size was small and the patients with SCI had different injury conditions and different patterns of hyperactive reflex dynamics. Further study should be conducted with more subjects involved.

Hyperactive reflexes in SCI were associated with significantly increased reflex gain, contraction rate, and decreased reflex threshold and reflex loop delay though there were different injury conditions within the spinal cord among those patients. With simplification, the methods described can potentially be used for neurological diagnosis and evaluations in clinical settings. Clinicians can potentially use it to evaluate hyperactive reflexes quantitatively with higher accuracy than the clinical DTR scale of 0 to 4. It can also be useful to evaluate treatment outcome more accurately. Clinicians could then track the outcome more accurately and make more accurate treatment planning. Practically, further work needs to be done to simplify the setup especially the tapping-induced output responses and substitute them with more convenient measures, such as reflex-mediated limb movement or tendon bounce-back force [21], which may make tendon reflex evaluations more conveniently done and suitable for clinical setting.

## Figures and Tables

**Table 1:** General information from SCI participants.

Subject	Age (year)	Gender	After injury (Year)	Level of injury	Complete or incomplete	DTR (0–4)	Ashworth scale (0–4)
A	27	M	3	T11	Incomplete	4	4
B	33	M	12	T7	Incomplete	4	4
C	38	M	10	C5	Incomplete	4	2
D	26	M	4	T1–T4	Incomplete	4	3
E	33	M	13	C5/6	Incomplete	2	2
F	29	M	4	L1	Incomplete	2	1
G	44	M	8	T6/7	Complete	3	2
H	25	M	1	T7	Complete	2	1
I	40	M	1	C5/C6	Complete	2	0
